# Admission nutritional-immunological indices and sepsis risk in emergency trauma patients: a retrospective cohort study

**DOI:** 10.3389/fnut.2026.1749592

**Published:** 2026-06-10

**Authors:** Zhenli Zhai, Wei Shen, Xue Fu, Yanqing Shi, Shimin Dong

**Affiliations:** 1 Department of Emergency, The Third Hospital of Hebei Medical University, Shijiazhuang, China; 2 Department of Anorectal Surgery, Handan Hospital of Traditional Chinese Medicine, Handan, China; 3 Department of Emergency, Handan Central Hospital, Handan, China

**Keywords:** CONUT score, emergency trauma, PNI, retrospective cohort analysis, sepsis

## Abstract

**Objective:**

This study aimed to investigate the association of the controlling nutritional status (CONUT) score and prognostic nutritional index (PNI) with the incidence of sepsis in emergency trauma patients, and to evaluate their potential clinical utility for the early prediction of sepsis.

**Materials and methods:**

The study enrolled trauma patients admitted to the Emergency Department of the Third Hospital of Hebei Medical University in China. CONUT score and PNI were calculated based on serum biomarkers to assess nutritional status. The primary outcome was the incidence of sepsis. Multivariate logistic regression analysis was performed to calculate adjusted odds ratios (OR) with 95% confidence intervals (CI). In addition, threshold effect analysis, subgroup analysis, and ROC curve analysis were conducted.

**Results:**

A total of 722 trauma patients admitted between January 1, 2021, and November 30, 2023, were included in the analysis. The incidence of sepsis was 33.1%. After adjusting for potential confounders, higher CONUT scores were significantly associated with an increased risk of sepsis (OR: 1.19, 95% CI: 1.04–1.36, *p* = 0.01), whereas higher PNI values were inversely associated with sepsis risk (OR: 0.88, 95% CI: 0.83–0.94, *p* < 0.001). Restricted cubic spline analysis demonstrated nonlinear associations of both CONUT and PNI with sepsis incidence. For CONUT <5, each 1-point increase was associated with a 2.43-fold higher odds of sepsis (OR: 2.43, 95% CI: 1.04–5.67, *p* = 0.0399); for PNI ≥ 39, each 1-unit increase was associated with a 35% lower odds of sepsis (OR: 0.65, 95% CI: 0.45–0.93, *p* = 0.0177). Subgroup analyses confirmed the robustness of these associations, with no significant interactions detected. Exploratory evaluation based on ROC curve analysis revealed that the AUCs of CONUT score and PNI for discriminating sepsis occurrence in trauma patients were 0.837 (95% CI: 0.808–0.866) and 0.851 (95% CI: 0.824–0.878), respectively.

**Conclusion:**

Both the CONUT score and PNI were independently associated with sepsis risk in emergency trauma patients and may be useful for early risk stratification. However, these findings are exploratory and require prospective, multicenter validation before clinical application.

## 1 Introduction

Trauma is the fourth leading cause of death worldwide. The resulting physical impairments, loss of labor capacity, and burden on healthcare and social resources far exceed those associated with most other diseases. Globally, severe trauma causes at least 5.8 million deaths each year (1). In China, trauma accounts for approximately 62 million medical visits annually and 700,000–800,000 fatalities, representing approximately 9% of all deaths (2).

Sepsis is one of the most common complications following trauma and a leading cause of in-hospital mortality among trauma patients (3). Its occurrence often prolongs intensive care unit (ICU) stays, thereby extending overall hospitalization and substantially increasing mortality. Therefore, early identification of patients at high risk for sepsis is critical for improving outcomes and reducing post-traumatic complications. Malnutrition is one of the key factors contributing to increased mortality in severe trauma patients (4). Previous studies have shown that malnutrition can nearly double the risk of death in critically injured trauma patients (5).

The CONUT score (6) (serum albumin, total cholesterol, lymphocyte count) and the PNI (7) (serum albumin, lymphocyte count) are composite indices of nutritional, inflammatory, and immunological status. These components are influenced not only by chronic nutritional reserves but also by acute post-traumatic responses: albumin, a negative acute-phase protein (8), may fall due to suppressed hepatic synthesis, capillary leak, and dilution from resuscitation fluids; lymphopenia can result from stress-hormone-mediated redistribution and sepsis-associated immune suppression (9); and total cholesterol often declines owing to reduced hepatic production and increased catabolism (10). Therefore, associations between CONUT/PNI and early post-traumatic sepsis carry both nutritional and acute-phase/immune implications. Although CONUT and PNI have been associated with outcomes in conditions such as cancer and heart failure (11–16), evidence in emergency trauma populations remains limited. We hypothesized that poorer admission nutritional-inflammatory-immunological status, reflected by higher CONUT scores and lower PNI values, would be independently associated with a higher incidence of sepsis within the first week after trauma.

## 2 Materials and methods

### 2.1 Study design and participants

This study was conducted in accordance with the Declaration of Helsinki, and all procedures involving patients were approved by the Ethics Committee of the Third Hospital of Hebei Medical University (Ethics Approval No: W2022-044-1). Due to the retrospective nature of the study, the requirement for informed consent was waived.

This retrospective cohort study included trauma patients admitted to the Emergency Department of the Third Hospital of Hebei Medical University, China, between January 1, 2021, and November 30, 2023. According to the Sepsis-3 criteria (17), patients who developed sepsis within 1 week after trauma were classified into the sepsis group, while those who did not were assigned to the non-sepsis group.

Inclusion criteria were as follows:

(a) Patients over the age of 18.
(b) No signs of infection (or suspected infection) prior to trauma.
(c) Admission within 1 week after injury.
(b) Hospital stay ≥24 h.
(e) Absence of end-stage liver/renal failure, malignant tumors, acquired immunodeficiency syndrome, or other malignant diseases.

Exclusion criteria were as follows:

(a) Patients under 18 years of age.
(b) Evidence or suspicion of pre-traumatic infection.
(c) Death within 24 h of admission.
(d) Missing data on either the CONUT score or PNI.
(e) Patients with end-stage liver/renal failure, malignant tumors, acquired immunodeficiency syndrome, or other malignant diseases.

### 2.2 Data collection

All data were collected through the electronic medical record system. Venous blood samples were collected within 24 h of admission. The first measurement result was selected for analysis. Serum albumin was measured using the bromocresol green method (Beckman Coulter AU5821), total cholesterol using the cholesterol oxidase method (Beckman Coulter AU5821), and lymphocyte counts were obtained using an automated hematology analyzer (Mindray BC-7500) from EDTA-anticoagulated whole blood. All assays were performed in accordance with the manufacturers’ instructions and the laboratory’s standard quality control procedures. As this was a retrospective study, fasting status could not be ascertained for all samples. The collected information included: (1) demographic characteristics: age, sex; (2) scoring systems (first complete score within 24 h of admission): Injury Severity Score (ISS), Sequential Organ Failure Assessment (SOFA) score, Glasgow Coma Scale (GCS) score; (3) comorbidities: diabetes mellitus; (4) laboratory parameters: white blood cell count (WBC), hemoglobin (Hb), platelet count (PLT), glucose (GLU), creatinine (CR), prothrombin time (PT) and D-dimer (D-D); (5) treatment interventions: mechanical ventilation (VENT) and emergency operation (EM operation); (6) complications: acute kidney injury (AKI), heart failure (HF), respiratory failure (RF), deep vein thrombosis (DVT), pneumonia and liver impairment (LIVER).

### 2.3 Calculation of CONUT score and PNI

The CONUT score was calculated as the sum of points assigned for serum albumin (0, 2, 4, 6 points), total cholesterol concentration, and total lymphocyte count (each scored 0, 1, 2, 3), as detailed in Table 1 (6). According to previously established CONUT score criteria, the study population was categorized into four groups: normal (0–1), mild risk (2–4), moderate risk (5–8) and severe risk (9–12).

**Table 1:** CONUT score calculation.

LYM (/mL)	Points	TC (mg/dL)	Points	ALB (g/dL)	Points
<0.8	3	<100	3	<2.5	6
0.8–1.1	2	100–139	2	2.5–2.9	4
1.2–1.6	1	140–180	1	3–3.5	2
>1.6	0	>180	0	>3.5	0

CONUT, controlling nutritional status; LYM, lymphocyte; TC, total cholesterol; ALB, albumin.

The PNI was calculated using the formula: PNI = 10 × serum albumin (g/dL) + 0.005 × total lymphocyte count (/mm^3^). Based on prior research (18), patients with a PNI < 39 were classified into the malnourished group, while those with a PNI ≥ 39 were classified into the normal nutrition group.

### 2.4 Outcomes

The outcome was the incidence of sepsis. Sepsis was defined as a suspected or confirmed infection accompanied by an increase in Sequential Organ Failure Assessment (SOFA) score of ≥2 points from baseline (17). To operationalize “suspected or confirmed infection” in this retrospective cohort, we used multiple sources of evidence: (1) clinical documentation in progress notes or discharge summaries indicating a confirmed or suspected infection; (2) initiation of a new systemic antimicrobial agent continued for ≥48 h (to exclude short-term prophylactic use); (3) microbiological evidence with positive cultures from blood or the relevant infection site; and/or (4) imaging or laboratory findings consistent with infection. A case was classified as having a suspected or confirmed infection if it met criterion (1) or (3), or if criterion (2) was present together with either (1) or (4).

### 2.5 Statistical analysis

Statistical analyses were performed using R version 4.2.2 (The R Foundation for Statistical Computing, http://www.R-project.org) and Free Statistics version 2.3, with statistical significance set at *p* < 0.05. Continuous variables were assessed for normality using the Shapiro–Wilk test. For comparisons between two independent groups, normally distributed variables were presented as mean ± standard deviation (SD) and analyzed using independent samples *t*-tests, while non-normally distributed variables were presented as median with interquartile range (IQR) and analyzed using the Mann–Whitney *U* test. Categorical variables were summarized as frequencies and percentages (%) and compared using Pearson’s χ^2^ test or Fisher’s exact test as appropriate.

Covariates for multivariate modeling were selected using a combined approach: expert judgment, existing literature, univariate association testing (*p* < 0.10 as screening threshold), clinical relevance, or a ≥10% change in effect estimates when the covariate was added. Multivariable logistic regression analysis was employed to evaluate the association between CONUT score, PNI and sepsis incidence. Different covariate adjustment models were constructed using an extended logistic modeling approach. Four regression models were specified: an unadjusted model; Model 1 adjusted for age, sex, ISS, SOFA and GCS score; Model 2 additionally included WBC, Hb, PLT, GLU, CR, PT, D-D, VENT and EM operation; Model 3 included adjustments for model 2 as well as AKI, HF, RF, DVT, pneumonia and LIVER.

Restricted cubic spline models with threshold analysis were applied to explore potential nonlinear relationships between CONUT score, PNI and sepsis risk. Subgroup analyses were conducted through stratification by relevant effect covariates. The ROC analysis was used for exploratory discriminative assessment of the study indices within the cohort.

## 3 Results

### 3.1 Baseline characteristics of patients

**Figure 1:**
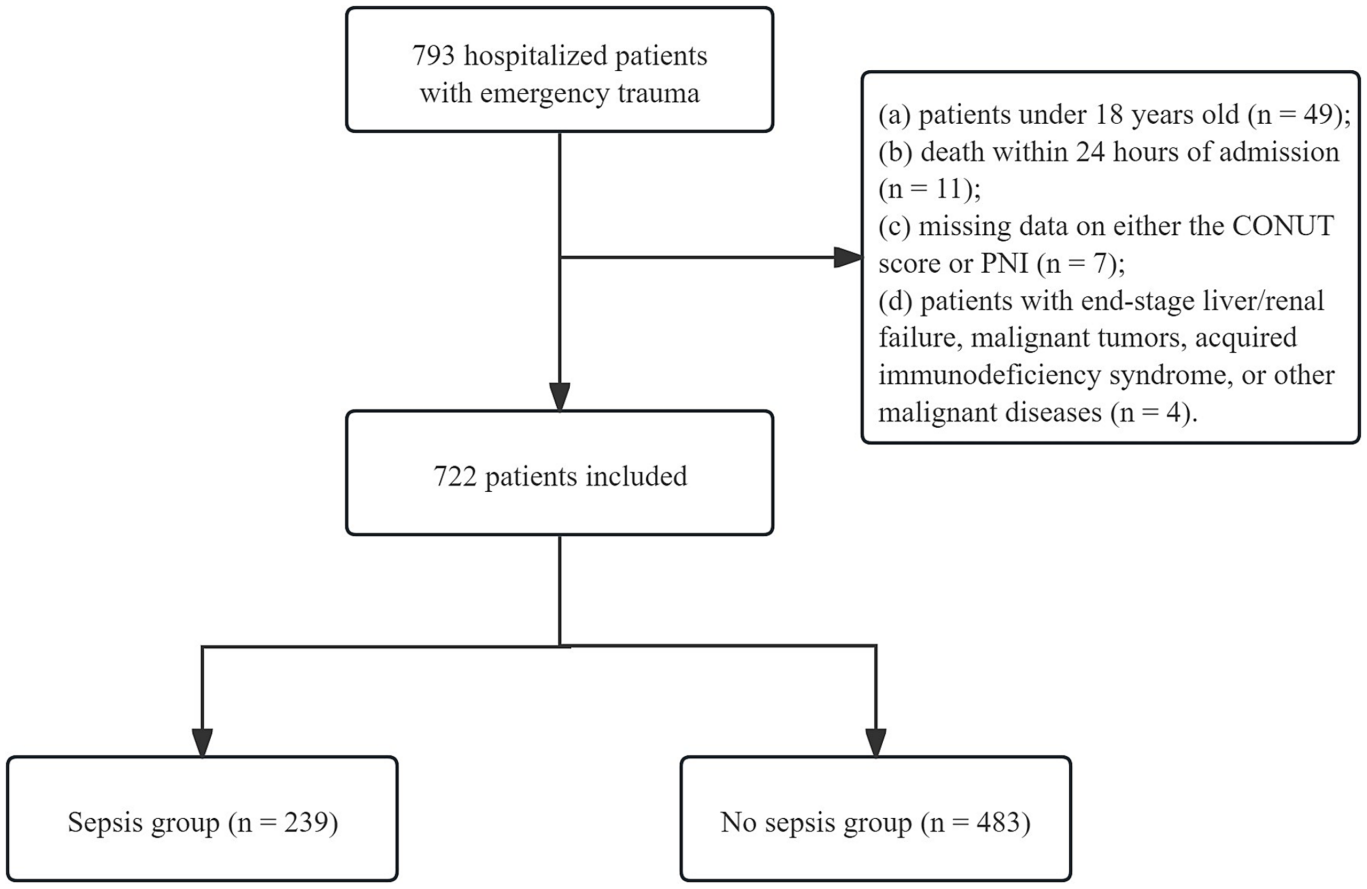
Flowchart in study participants. CONUT, controlling nutritional status; PNI, prognostic nutritional index.

Initially, 793 trauma patients admitted via the emergency department were enrolled in the study. After the inclusion/exclusion process, 71 patients were excluded, including 7 due to missing CONUT score or PNI data. The final analysis cohort consisted of 722 patients with complete data for all study variables (Figure 1); no imputation was performed (complete-case analysis).

Among them, 239 patients were in the sepsis group, corresponding to an overall incidence of 33.1%. Males accounted for 76.6% of the sepsis group (Table 2). Significant differences were observed between the sepsis and non-sepsis groups in terms of age, sex, ISS, GCS, SOFA, CONUT, PNI, VENT and EM operation (*p* < 0.05). In contrast, no statistically significant differences were found in comorbidities and complications, such as diabetes, liver disease, deep vein thrombosis and pneumonia (*p* > 0.05).

**Table 2:** Baseline characteristics stratified by incidence of sepsis.

Variables	Total (*n* = 722)	Sepsis (*n* = 239)	Non-sepsis (*n* = 483)	*p*-value
Age (year)	51.2 ± 14.7	55.5 ± 14.1	49.0 ± 14.5	<0.001
Male, *n* (%)	515 (71.3)	183 (76.6)	332 (68.7)	0.029
ISS	13.0 (4.0, 29.0)	34.0 (25.0, 41.0)	8.0 (4.0, 13.5)	<0.001
GCS	12.9 ± 4.0	9.8 ± 4.9	14.4 ± 2.3	<0.001
SOFA	1.0 (0.0, 5.0)	6.0 (4.0, 8.0)	1.0 (0.0, 2.0)	<0.001
WBC (×10^9^/L)	9.7 ± 3.9	10.6 ± 4.4	9.2 ± 3.5	<0.001
Hb (g/L)	113.8 ± 24.8	99.9 ± 21.2	120.7 ± 23.6	<0.001
PLT (×10^9^/L)	171.0 ± 75.0	128.5 ± 72.1	192.1 ± 67.1	<0.001
GLU (mmol/L)	6.9 (5.8, 8.5)	8.0 (6.9, 9.4)	6.3 (5.7, 7.7)	<0.001
Cr (μmol/L)	74.2 ± 37.6	90.8 ± 54.3	65.9 ± 21.3	<0.001
PT (s)	14.4 ± 3.2	15.0 ± 2.5	14.0 ± 3.4	<0.001
D-Dimmer (μg/ml)	3.0 (1.3, 8.7)	7.4 (3.1, 15.9)	2.2 (1.0, 5.4)	<0.001
Diabetes, *n* (%)	71 (9.8)	22 (9.2)	49 (10.1)	0.69
EM operation, *n* (%)	121 (16.8)	77 (32.2)	44 (9.1)	<0.001
VENT, *n* (%)	222 (30.7)	190 (79.5)	32 (6.6)	<0.001
LIVER, *n* (%)	176 (24.4)	58 (24.3)	118 (24.4)	0.962
DVT, *n* (%)	239 (33.1)	76 (31.8)	163 (33.7)	0.601
AKI, *n* (%)	73 (10.1)	22 (9.2)	51 (10.6)	0.57
HF, *n* (%)	82 (11.4)	24 (10)	58 (12)	0.433
Pneumonia, *n* (%)	236 (32.7)	75 (31.4)	161 (33.3)	0.599
RF, *n* (%)	167 (23.1)	56 (23.4)	111 (23)	0.893
CONUT	4.0 (2.0, 7.0)	7.0 (5.0, 10.0)	3.0 (1.0, 4.5)	<0.001
CONUT (categorical), *n* (%)				<0.001
Normal	143 (19.8)	2 (0.8)	141 (29.2)	
Mild risk	266(36.8)	45 (18.8)	221 (45.8)	
Moderate risk	182 (25.2)	107 (44.8)	75 (15.5)	
Severe risk	131 (18.1)	85 (35.6)	46 (9.5)	
PNI	40.4 ± 9.5	33.0 ± 7.1	44.1 ± 8.4	<0.001
PNI (categorical), *n* (%)				<0.001
<39	305 (42.2)	191 (79.9)	114 (23.6)	
≥39	417 (57.8)	48 (20.1)	369 (76.4)	

Date presented are *n* (%) or mean ± SD or median (quartile).

ISS, injury severity score; GCS, glasgow coma scale; SOFA, sequential organ failure assessment; WBC, white blood cell; Hb, hemoglobin; PLT, platelet; GLU, glucose; Cr, creatinine; PT, prothrombin time; EM operation, emergency operation; VENT, mechanical ventilation; LIVER, chronic liver disease; DVT, deep venous thrombosis; AKI, acute kidney injury; HF, heart failure; RF, respiratory failure; CONUT, controlling nutritional status; PNI, Prognostic Nutritional Index.

### 3.2 Multivariate logistic regression analysis

Detailed results of the univariate logistic regression analysis are presented in Supplementary Table S1. Age, ISS, SOFA, WBC, CR, GLU, PT, D-D, VENT, EM operation, and CONUT score (including its categorical form) were identified as risk factors for sepsis incidence (*p* < 0.05). Female, GCS, Hb, PLT, and PNI (including the ≥39 category) were identified as protective factors (*p* < 0.05). Diabetes, DVT and pneumonia showed no significant association with sepsis incidence (*p* > 0.05).

We constructed three multivariable logistic regression models to assess the independent effects of the CONUT score and PNI on sepsis incidence (Table 3). Odds ratios (ORs) with 95% confidence intervals (CIs) are shown in Table 3. Both the CONUT score and PNI demonstrated robust associations with sepsis across the unadjusted model and all three adjusted models (*p* < 0.05). In the unadjusted model, each 1-point increase in the CONUT score was associated with a 46% higher risk of sepsis (OR: 1.46, 95% CI: 1.38–1.55, *p* < 0.001). Conversely, each 1-unit increase in PNI was associated with a 15% reduction in sepsis risk (OR: 0.85, 95% CI: 0.82–0.87, *p* < 0.001). After sequential adjustment for confounders, the effect size for the CONUT score in Model 3 indicated a 19% increased risk per 1-point increment (OR: 1.19, 95% CI: 1.04–1.36, *p* = 0.01); for the PNI, the adjusted Model 3 showed a 12% decreased risk per 1-unit increase (OR: 0.88, 95% CI: 0.83–0.94, *p* < 0.001).

**Table 3:** The association between CONUT score and PNI and the incidence of sepsis.

Variables	Unadjusted		Model 1		Model 2		Model 3	
	OR (95% CI)	*p*	OR (95% CI)	*p*	OR (95% CI)	*p*	OR (95% CI)	*p*
CONUT	1.46 (1.38–1.55)	<0.001 <0.001	1.10 (1.01–1.19)	0.025 <0.001	1.15 (1.01–1.31) 1.16 (1.02 ~ 1.32)	0.031	1.19 (1.04–1.36)	0.010
CONUT (categorical)								
Normal	1(Ref)		1(Ref)		1(Ref)		1(Ref)	
Mild risk	14.36 (3.43–60.06)	<0.001	7.07 (1.47–34.07)	0.015 <0.001	9.71 (1.45–65.14)	0.019	10.45 (1.51–72.52)	0.018
Moderate risk	100.58 (24.17–418.54)	<0.001	16.32 (3.41–78.17)	<0.001	22.64 (3.25–157.7) (11.51 ~ 221.57) (11.51 ~ 221.57)	0.002	28.07 (3.86–203.94)	0.001
Severe risk	130.27 (30.86–550.01)	<0.001	7.12 (1.43–35.44.49)	0.017 <0.001	12.97 (1.62–103.66)	0.016	17.82 (2.11–150.46)	0.008
*p* for trend		<0.001		0.036 <0.001		0.030		0.010
PNI	0.85 (0.82–0.87)	<0.001	0.95 (0.92–0.98)	0.003	0.90 (0.84–0.95)	<0.001	0.88 (0.83–0.94)	< 0.001
PNI (categorical)								
<39	1(Ref)		1(Ref)		1(Ref)		1(Ref)	
≥39	0.08 (0.05–0.11)	<0.001	0.37 (0.18–0.80)	0.011	0.38 (0.18–0.80)	<0.001	0.31 (0.14–0.68)	0.003

Model 1: Adjusted for age, sex, ISS, SOFA, GCS. Model 2: Model 1 + WBC, Hb, PLT, GLU, Cr, PT, D-D, VENT, EM operation. Model 3: Model 2 + AKI, HF, RF, DVT, Pneumonia, Liver, diabetes. OR, odds ratio; CI, confidence interval; ISS,injury severity score; GCS, glasgow coma scale; SOFA, sequential organ failure assessment; WBC, white blood cell; Hb, hemoglobin; PLT, platelet; GLU, glucose; Cr, creatinine; PT, prothrombin time; EM operation, emergency operation; VENT, Mechanical ventilation; LIVER, chronic liver disease; DVT, deep venous thrombosis; AKI, acute kidney injury; HF, heart failure; RF, respiratory failure; CONUT, controlling nutritional status; PNI, prognostic nutritional index.

For additional analysis, continuous variables were converted to categorical forms. Using the normal CONUT group as the reference, both the moderate risk group (OR: 28.07, 95% CI: 3.86–203.94, *p* = 0.001) and the severe risk group (OR: 17.82, 95% CI: 2.11–150.46, *p* = 0.008) showed significantly increased odds of sepsis. In the categorical analysis of PNI, using the “PNI < 39” group as reference, the “PNI ≥ 39” group in Model 3 showed significantly lower odds of sepsis (OR: 0.31, 95% CI: 0.14–0.68, *p* = 0.003).

### 3.3 Restricted cubic splines analysis

**Figure 2:**
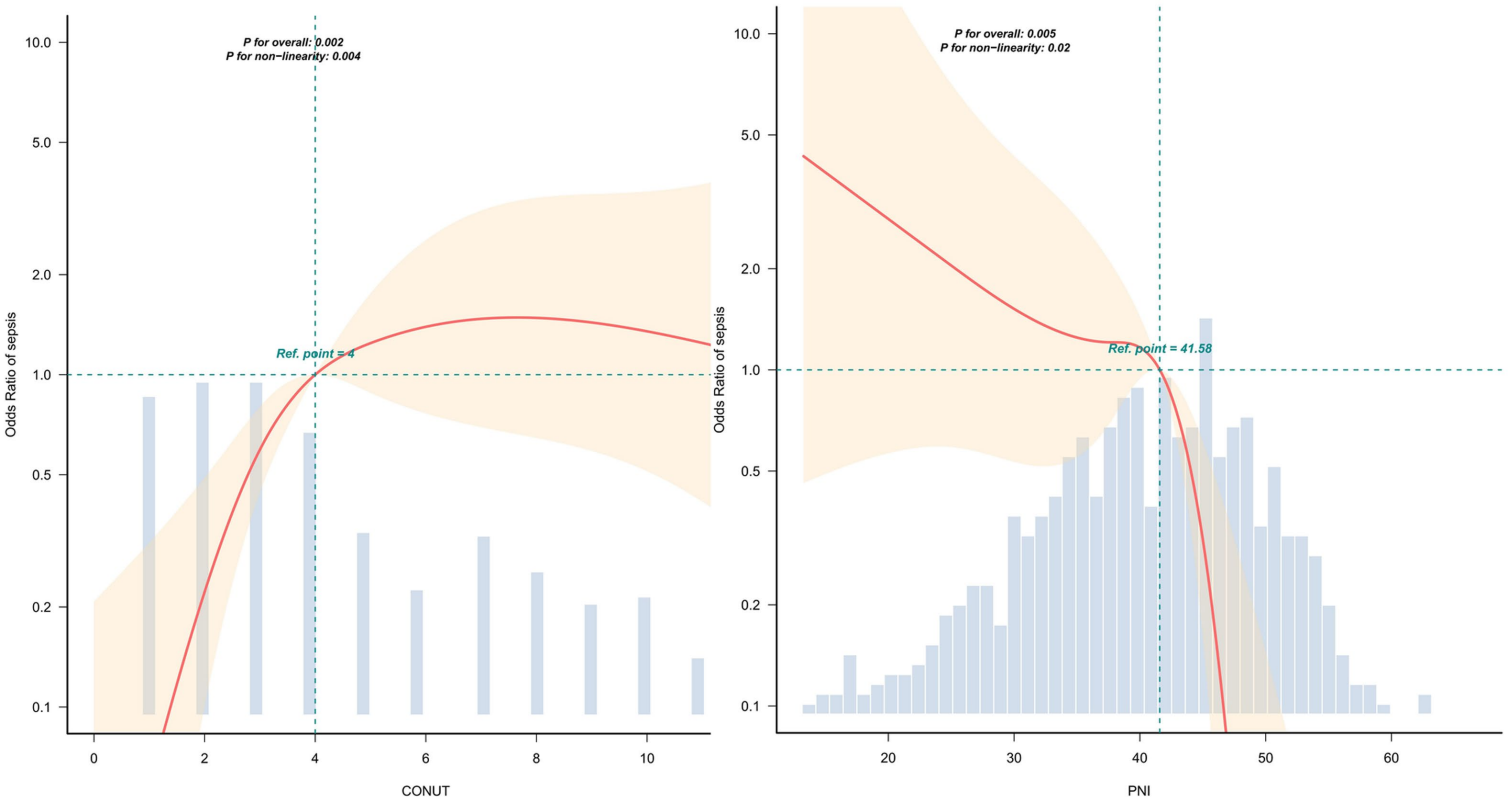
Restrictive cubic spline analysis for the association between CONUT score and PNI and the sepsis incidence. Adjusted for age, sex, ISS, SOFA, GCS, WBC, Hb, PLT, GLU, CR, PT, D-D, VENT, EM operation, AKI, HF, RF, DVT, Pneumonia, Liver, diabetes. OR, odds ratio; CI, confidence interval; ISS, injury severity score; GCS, glasgow oma cale; SOFA, sequential organ failure assessment; WBC, white blood cell; Hb, hemoglobin; PLT, platelet; GLU, glucose; Cr, creatinine; PT, prothrombin time; EM operation, emergency operation; VENT, echanical ventilation; LIVER, chronic liver disease; DVT, deep venous thrombosis; AKI, acute kidney injury; HF, heart failure; RF, respiratory failure; CONUT, controlling nutritional status; PNI, prognostic nutritional index.

Restricted cubic spline (RCS) analysis revealed a nonlinear dose–response relationship between CONUT score and sepsis incidence after adjusting for relevant confounders (*p* = 0.004) (Figure 2; Table 4). Threshold analysis identified an inflection point at a CONUT score of 5.0. Below this threshold, there was a significant positive association with sepsis risk (OR: 2.344, 95% CI: 1.059–5.186; *p* = 0.0356). However, no significant association was observed above the inflection point (OR: 1.056, 95% CI: 0.874–1.275; *p* = 0.5756).

**Table 4:** Threshold effect analysis of CONUT score on sepsis incidence.

Item	Sepsis incidence	*p*-value
CONUT score	OR (95%CI)	
<5.0	2.344 (1.059–5.186)	0.0356
≥5.0	1.056 (0.874–1.275)	0.5756
Likelihood Ratio test	—	< 0.001

Adjusted for age, sex, ISS, SOFA, GCS, WBC, Hb, PLT, GLU, CR, PT, D-D, VENT, EM operation, AKI, HF, RF, DVT, Pneumonia, Liver, diabetes. OR, odds ratio; CI, confidence interval; ISS, injury severity score; GCS, glasgow coma scale; SOFA, sequential organ failure assessment; WBC, white blood cell; Hb, hemoglobin; PLT, platelet; GLU, glucose; Cr, creatinine; PT, prothrombin time; EM operation, emergency operation; VENT, Mechanical ventilation; LIVER, chronic liver disease; DVT, deep venous thrombosis; AKI, acute kidney injury; HF, heart failure; RF, respiratory failure; CONUT, controlling nutritional status; PNI, prognostic nutritional index.

Similarly, a nonlinear relationship was found between PNI and sepsis incidence after confounder adjustment (*p* = 0.02) (Figure 2; Table 5). The two-piecewise linear regression model identified an inflection point at a PNI value of 39. No significant association was detected on the left side of the threshold (OR: 0.942, 95% CI: 0.857–1.035; *p* = 0.2148). In contrast, on the right side of the inflection point, each unit increase in PNI was associated with a significant 34% reduction in sepsis risk (OR: 0.66, 95% CI: 0.0463–0.94; *p* = 0.0212).

**Table 5:** Threshold effect analysis of PNI score on sepsis incidence.

Item	Sepsis incidence	*p*-value
PNI score	OR (95%CI)	
<39	0.942 (0.857–1.035)	0.2148
≥39	0.66 (0.463–0.94)	0.0212
Likelihood ratio test	—	<0.001

Adjusted for age, sex, ISS, SOFA, GCS, WBC, Hb, PLT, GLU, CR, PT, D-D, VENT, EM operation, AKI, HF, RF, DVT, Pneumonia, Liver, diabetes. OR, odds ratio; CI, confidence interval; ISS, injury severity score; GCS, glasgow coma scale; SOFA, sequential organ failure assessment; WBC, white blood cell; Hb, hemoglobin; PLT, platelet; GLU, glucose; Cr, creatinine; PT, prothrombin time; EM operation, emergency operation; VENT, Mechanical ventilation; LIVER, chronic liver disease; DVT, deep venous thrombosis; AKI, acute kidney injury; HF, heart failure; RF, respiratory failure; CONUT, controlling nutritional status; PNI, prognostic nutritional index.

### 3.4 Subgroup analyses

**Figure 3:**
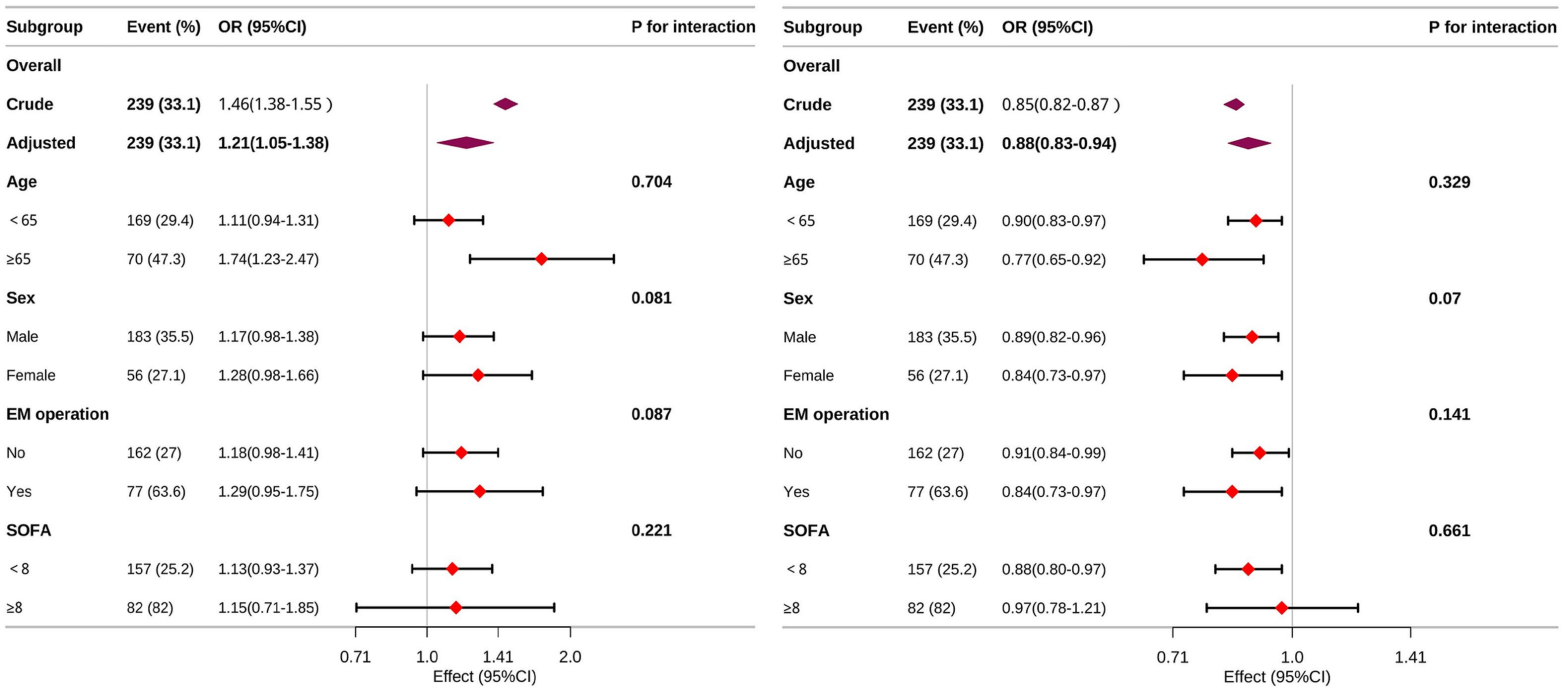
Subgroup analysis of association between CONUT score and PNI and incidence of sepsis. Adjusted for age, sex, ISS, SOFA, GCS, WBC, Hb, PLT, GLU, CR, PT, D-D, VENT, EM operation, AKI, HF, RF, DVT, pneumonia, liver, diabetes. OR, odds ratio; CI, confidence interval; ISS, injury severity score; GCS, Glasgow coma scale; SOFA, sequential organ failure assessment; WBC, white blood cell; Hb, hemoglobin; PLT, platelet; GLU, glucose; Cr, creatinine; PT, prothrombin time; EM operation, emergency operation; VENT, Mechanical ventilation;, chronic liver disease; DVT, deep venous thrombosis; AKI, acute kidney injury; HF, heart failure; RF, respiratory failure; CONUT, controlling nutritional status; PNI, prognostic nutritional index.

Subgroup analyses stratified by age, sex, EM operation, and SOFA score demonstrated consistent associations between both CONUT score and PNI with sepsis incidence, showing no significant interaction effects (Figure 3).

### 3.5 ROC curve analysis

**Figure 4:**
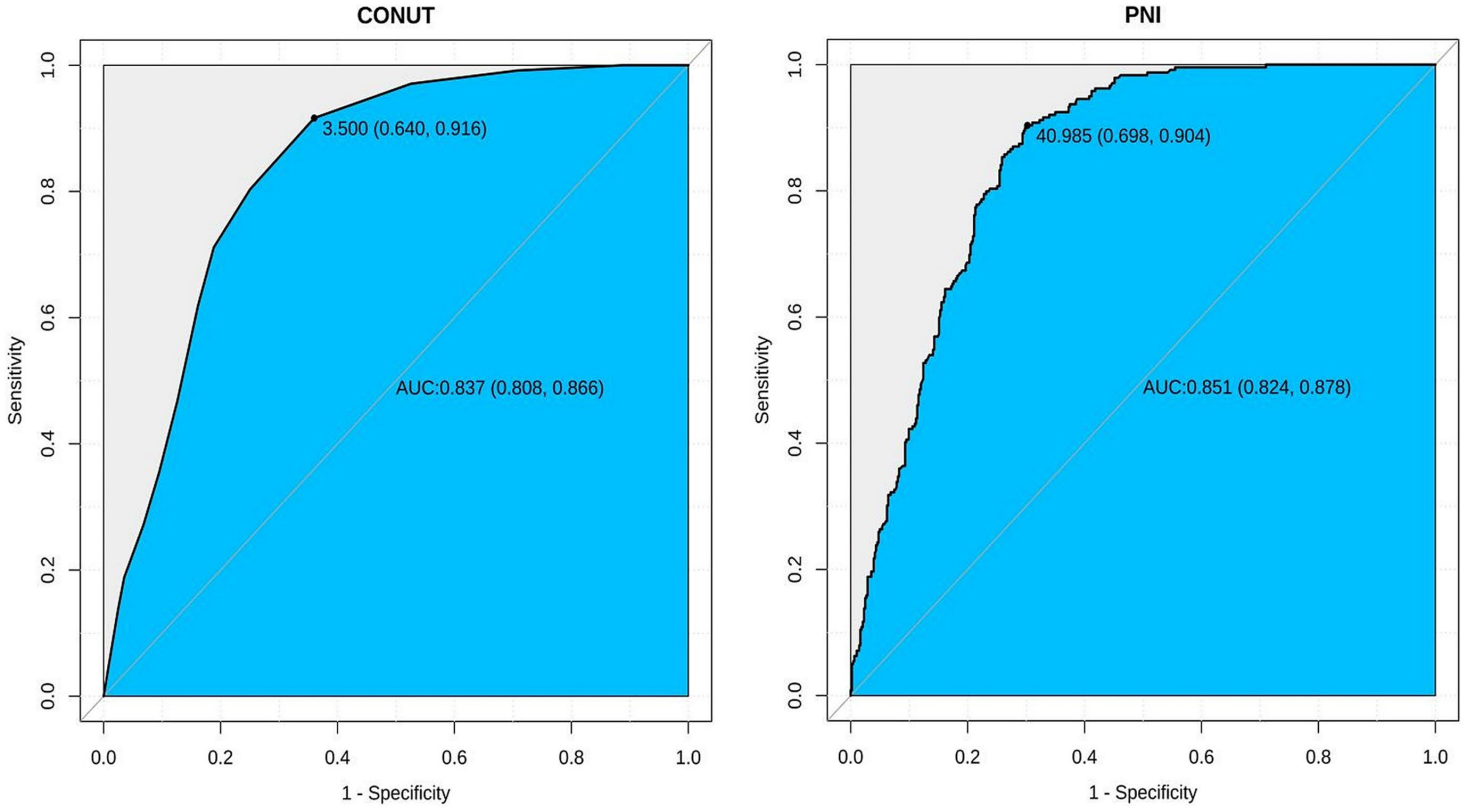
ROC curve of CONUT score and PNI predicting in incidence of sepsis by trauma patients. CONUT, controlling nutritional status; PNI, prognostic nutritional index.

ROC curve analysis was performed to exploratory discriminative assessment of *CONUT and PNI* for sepsis incidence in trauma patients. The results indicated that both nutritional assessment tools demonstrated predictive value. The CONUT score yielded an AUC of 0.837 (95% CI: 0.808–0.866), with an optimal cut-off value of 3.5 providing a sensitivity of 90.63% and a specificity of 63.98% for predicting sepsis. The PNI showed an AUC of 0.851 (95% CI: 0.824–0.878), with a cut-off value of 40.985 providing a sensitivity of 90.38% and a specificity of 69.77% (Figure 4; Table 6). The ROC curves showed that both had good discriminative ability, and bootstrap internal validation confirmed the stability of the results (Supplementary Figure S1).

**Table 6:** Risk stratification value of parameters for sepsis in patients with trauma.

Parameter	AUC	95% CI	Youden index	Cut off value	Sensitivity (%)	Specificity (%)
CONUT score	0.837	0.808–0.866	0.6015	3.5	91.63	63.98
PNI	0.851	0.824–0.878	0.5561	40.985	90.38	69.77

CONUT, controlling nutritional status; PNI, prognostic nutritional index; CI, confidence interva.

### 3.6 Sensitivity analyses

Sensitivity analyseswere performed by: (1) excluding the 16 patients with time from injury to admission >72 h (Supplementary Table S2), (2) excluding the SOFA covariate (Supplementary Table S3), (3) excluding comorbidities and complications (Supplementary Table S4), and (4) combining the normal and mild groups as the reference category (Supplementary Table S5). After each sensitivity analysis, the direction and significance of the primary effect estimates did not change materially.

## 4 Discussion

In this single-center retrospective cohort study of emergency trauma patients, we systematically evaluated the association between two nutritional indices (CONUT and PNI) and sepsis incidence. The key findings are as follows. First, the prevalence of malnutrition risk varied substantially by assessment tool: CONUT identified nutritional risk in 80.1% of patients (mild 36.8%, moderate 25.2%, severe 18.1%), whereas PNI classified 42.2% as malnourished-a proportion closely matching the combined CONUT moderate and severe categories (43.3%). This suggests that CONUT is well suited for broad screening, while PNI more specifically identifies moderate or greater malnutrition, supporting a complementary use of the two tools. Second, after adjustment for confounders, both CONUT and PNI remained significantly associated with sepsis. Restricted cubic spline (RCS) analysis revealed nonlinear associations: for CONUT, an inflection point at 5.0 (below 5.0, each unit increase was associated with substantially higher sepsis odds; above 5.0 the association plateaued and was non-significant); for PNI, an inflection point at 39.0 (values ≥39 were associated with progressively lower sepsis risk per unit increase, while PNI < 39 showed no significant association). ROC analysis gave optimal cut-offs of CONUT 3.5 (sensitivity 90.63%, specificity 63.98%) and PNI 41.985 (sensitivity 90.38%, specificity 69.77%). Both indices showed good discriminative ability (CONUT AUC 0.837, PNI AUC 0.851) with bootstrap-confirmed stability. The directionality and significance were consistent across prespecified subgroups, supporting their value for early risk stratification.

Trauma can disrupt immune function, reduce resistance, and increase susceptibility to complications such as sepsis or multiple organ dysfunction, thereby elevating mortality risk. Our observed sepsis incidence of 33.1% is consistent with previous reports (19). Despite continuous advancements in medical technology (20–22), the risk of secondary sepsis in trauma patients remains high, underscoring the importance of early risk prediction. The CONUT score and PNI are novel nutritional-inflammatory composite indices that are simple, low-cost, and easily applicable as immunonutrition screening tools. Unlike ICU-oriented tools such as NUTRIC/mNUTRIC (which incorporate illness severity and comorbidity burden to guide nutritional therapy in critically ill patients), CONUT and PNI rely solely on routine laboratory tests and are therefore more suitable for early screening in emergency and trauma settings. The CONUT score, which integrates serum albumin, total lymphocyte count, and total cholesterol, is used to assist in nutritional status assessment (23, 24). In this scoring system, lower levels of each component result in a higher total score, implying poorer nutritional status (25). This scale has been widely used as an indicator of disease severity and mortality across various conditions, particularly in gastrointestinal cancer and cardiac diseases (26–30). Recent studies have confirmed the prognostic value of PNI in multiple malignancies (31, 32), COVID-19 (33, 34), and coronary artery disease (35); however, research on its prognostic value in emergency trauma patients remains limited. Our study extends this evidence by demonstrating that both CONUT and PNI are independently associated with post-trauma sepsis.

Pathophysiologically, the abnormalities captured by these indices - hypoalbuminemia, lymphopenia, and low cholesterol-reflect diminished protein/caloric reserves and impaired cellular immunity, mechanisms that plausibly increase susceptibility to infection and progression to sepsis, particularly after major trauma. It is critically important to recognize that in the acute trauma setting, low albumin is not equivalent to chronic malnutrition. Instead, trauma rapidly induces a state of hypermetabolism and hypercatabolism, leading to sharply increased consumption of energy and protein (36). More specifically, hypoalbuminemia in polytrauma patients arises from multiple intertwined mechanisms: (a) systemic inflammation (pro-inflammatory cytokines such as IL-6 and TNF-αsuppress albumin synthesis); (b) capillary leakage (albumin extravasates from the vascular space into the interstitial compartment); (c) fluid resuscitation (large volumes of crystalloids cause hemodilution); and (d) the acute-phase response (albumin is a negative acute-phase protein, down-regulated during stress). Similarly, lymphopenia reflects the post-traumatic immunosuppression state, with lymphocytes mediating adaptive immunity and providing regulatory and protective functions; a low lymphocyte count typically indicates impaired immune status, thereby increasing the risk of secondary infections and sepsis (37). Low cholesterol in the CONUT score further points to depleted caloric intake and energy reserves. Therefore, rather than being merely a marker of isolated chronic malnutrition, the CONUT score -through serum albumin, cholesterol, and lymphocyte count -objectively reflects the severity of post-traumatic biological vulnerability that integrates nutritional depletion, inflammation, and immune dysfunction. Likewise, PNI (combining albumin and lymphocyte count) directly captures both the nutritional and immune defense dimensions.

Our findings provide new evidence for clinical risk stratification and individualized treatment in emergency trauma patients. The observed threshold-dependent relationships (RCS inflection points and ROC-derived cut-offs) should be considered exploratory and cohort-specific; nonetheless, the consistent directionality across subgroups supports the use of CONUT and PNI as early, admission-time biomarkers for sepsis risk. Because both indices rely on routine blood tests (albumin, lymphocyte count, cholesterol), they can be rapidly obtained at no additional cost, facilitating early identification of high-risk patients who may benefit from targeted interventions such as enhanced monitoring, infection prevention measures, or immunonutrition support. The complementary nature of CONUT (broader screening) and PNI (more specific for moderate/severe malnutrition) may guide a two-step assessment strategy in the emergency setting.

This study has several limitations. First, as a single-center retrospective analysis, it is susceptible to selection bias and has limited external generalizability. Second, although we adjusted for multiple covariates in multivariable regression, residual confounding, suboptimal variable selection, or model overfitting cannot be entirely excluded; moreover, the use of complete-case analysis to exclude subjects with missing data may have introduced additional selection bias. Third, the nutritional indices were derived from blood samples collected within 24 h of admission and should be regarded as early, admission-time risk-stratification biomarkers; they may not reflect pre-injury chronic nutritional status or temporal in-hospital changes. Fourth, restricting the primary outcome to sepsis occurring within 1 week after injury helps focus on events most plausibly related to the index trauma and reduces temporal confounding from later exposures, but it may miss late-onset sepsis, underestimate overall incidence, bias findings toward early severe cases, and preclude assessment of longer-term outcomes. Fifth, the threshold effects (inflection points and ROC cut-offs) are exploratory and cohort-specific; they require external validation before clinical implementation.

Future studies should be prospective, multicenter, and include longer follow-up with time-to-event and sensitivity analyses, serial measurements of nutritional and inflammatory parameters, detailed in-hospital exposure data (e.g., antibiotics, procedures, surgeries), and more comprehensive covariates to validate and refine these findings. External validation cohorts are needed to confirm the generalizability of the identified thresholds. Additionally, interventional studies examining whether early immunonutrition support in patients with high CONUT or low PNI can reduce sepsis incidence would be of great clinical relevance.

## 5 Conclusion

This study found that both the CONUT score and PNI were independently associated with sepsis incidence in emergency trauma patients. Our RCS analysis identified nonlinear relationships and potential thresholds that may inform future research, but these findings are exploratory and require external and prospective validation before they can inform clinical management thresholds or interventions.

## Data Availability

The raw data supporting the conclusions of this article will be made available by the authors, without undue reservation.

## References

[1] GBD 2017 Causes of Death Collaborators. Global, regional, and national age-sex-specific mortality for 282 causes of death in 195 countries and territories, 1980-2017: a systematic analysis for the global burden of disease study 2017. Lancet. (2018) 392:1736–88. doi: 10.1016/S0140-6736(18)32203-7 30496103 PMC6227606

[2] Zhou J Wang T Belenkiy I Hardcastle TC Rouby JJ Jiang B . Management of severe trauma worldwide: implementation of trauma systems in emerging countries: China, Russia and South Africa. Crit Care. (2021) 25:286. doi: 10.1186/s13054-021-03681-8, 34372903 PMC8352140

[3] Eidelman LA Putterman D Putterman C Sprung CL. The spectrum of septic encephalopathy. Definitions, etiologies, and mortalities. JAMA. (1996) 275:470–3. doi: 10.1001/jama.1996.03530300054040 8627969

[4] Dijkink S Meier K Krijnen P Yeh DD Velmahos GC Schipper IB. Malnutrition and its effects in severely injured trauma patients. Eur J Trauma Emerg Surg. (2020) 46:993–1004. doi: 10.1007/s00068-020-01304-5, 31974669 PMC7593306

[5] Ceniccola GD Okamura AB Sepúlveda Neta JDS Lima FC Santos Deus AC Oliveira JA Association between AND-ASPEN malnutrition criteria and hospital mortality in critically ill trauma patients: a prospective cohort study JPEN J Parenter Enteral Nutr (2020) 44 1347–1354 doi: 10.1002/jpen.1795 32026492

[6] de Ignacio Ulíbarri J González-Madroño A de Villar NG González P González B Mancha A . CONUT: a tool for controlling nutritional status. First validation in a hospital population. Nutr Hosp. (2005) 20:38–45. 15762418

[7] Shi Y Wang Y Sun T Du L Lv Y Chen Z . A novel nutritional immune risk score model for long-term prognosis in colorectal cancer using clustering and principal component analysis. Front Nutr. (2026) 13:1734873. doi: 10.3389/fnut.2026.1734873, 42063957 PMC13124479

[8] Rubio-Baines I Camporota L González-Delgado D Echarri G Sala-Trull MC Montero-López P . Use of human serum albumin in critically ill patients: a narrative review. J Clin Med. (2026) 15:1981. doi: 10.3390/jcm15051981, 41827398 PMC12986204

[9] Yang J Ma B Tong H. Lymphocyte count trajectories are associated with the prognosis of sepsis patients. Crit Care. (2024) 28:399. doi: 10.1186/s13054-024-05186-6, 39623501 PMC11613510

[10] Buzby GP Mullen JL Matthews DC Hobbs CL Rosato EF. Prognostic nutritional index in gastrointestinal surgery. Am J Surg. (1980) 139:160–7. doi: 10.1016/0002-9610(80)90246-9, 7350839

[11] Greenberg JA Hsu J Bawazeer M Marshall J Friedrich JO Nathens A . Clinical practice guideline: management of acute pancreatitis. Can J Surg. (2016) 59:128–40. doi: 10.1503/cjs.015015, 27007094 PMC4814287

[12] Wang X Cui Z Li H Saleen AF Zhang D Miao B . Nosocomial mortality and early prediction of patients with severe acute pancreatitis. J Gastroenterol Hepatol. (2010) 25:1386–93. doi: 10.1111/j.1440-1746.2010.06376.x, 20659228

[13] Cortes VA Busso D Maiz A Arteaga A Nervi F Rigotti A. Physiological and pathological implications of cholesterol. Front Biosci. (2014) 19:416–28. doi: 10.2741/4216, 24389193

[14] Soeters PB Wolfe RR Shenkin A. Hypoalbuminemia: pathogenesis and clinical significance. JPEN J Parenter Enteral Nutr. (2019) 43:181–93. doi: 10.1002/jpen.1451, 30288759 PMC7379941

[15] Lo Buglio A Bellanti F Capurso C Vendemiale G. Controlling nutritional status (CONUT) score as a predictive marker in hospitalized frail elderly patients. J Pers Med. (2023) 13:1119. doi: 10.3390/jpm13071119, 37511732 PMC10381597

[16] Hong W Zimmer V Basharat Z Zippi M Stock S Geng W . Association of total cholesterol with severe acute pancreatitis: a U-shaped relationship. Clin Nutr. (2020) 39:250–7. doi: 10.1016/j.clnu.2019.01.022, 30772093

[17] Singer M Deutschman CS Seymour CW Shankar-Hari M Annane D Bauer M . The third international consensus definitions for sepsis and septic shock (sepsis-3). JAMA. (2016) 315:801–10. doi: 10.1001/jama.2016.0287, 26903338 PMC4968574

[18] Raposeiras Roubín S Abu Assi E Cespón Fernandez M Barreiro Pardal C Lizancos Castro A Parada JA . Prevalence and prognostic significance of malnutrition in patients with acute coronary syndrome. J Am Coll Cardiol. (2020) 76:828–40. doi: 10.1016/j.jacc.2020.06.058, 32792081

[19] Mas-Celis F Olea-López J Parroquin-Maldonado JA. Sepsis in trauma: a deadly complication. Arch Med Res. (2021) 52:808–16. doi: 10.1016/j.arcmed.2021.10.007, 34706851

[20] Oyeniyi BT Fox EE Scerbo M Tomasek JS Wade CE Holcomb JB. Trends in 1029 trauma deaths at a level 1 trauma center: impact of a bleeding control bundle of care. Injury. (2017) 48:5–12. doi: 10.1016/j.injury.2016.10.037, 27847192 PMC5193008

[21] Eguia E Bunn C Kulshrestha S Zern N Han J Hekman D . Trends, cost, and mortality from sepsis after trauma in the United States: an evaluation of the national inpatient sample of hospitalizations, 2012–2016. Crit Care Med. (2020) 48:1296–303. doi: 10.1097/CCM.0000000000004451 32590387 PMC7872079

[22] Wafaisade A Lefering R Bouillon B Sakka SG Thamm OC Paffrath T . Epidemiology and risk factors of sepsis after multiple trauma: an analysis of 29,829 patients from the trauma registry of the German Society for Trauma Surgery. Crit Care Med. (2011) 39:621–8. doi: 10.1097/CCM.0b013e318206d3df, 21242798

[23] Li W Li M Wang T Ma G Deng Y Pu D . Controlling nutritional status (CONUT) score is a prognostic factor in patients with resected breast cancer. Sci Rep. (2020) 10:6633. doi: 10.1038/s41598-020-63610-7, 32313183 PMC7171067

[24] Hayama T Ozawa T Okada Y Tsukamoto M Fukushima Y Shimada R . The pretreatment controlling nutritional status (CONUT) score is an independent prognostic factor in patients undergoing resection for colorectal cancer. Sci Rep. (2020) 10:13239. doi: 10.1038/s41598-020-70252-2, 32764671 PMC7413386

[25] Sargento L Longo S Lousada N Dos Reis RP. The importance of assessing nutritional status in elderly patients with heart failure. Curr Heart Fail Rep. (2014) 11:220–6. doi: 10.1007/s11897-014-0189-5, 24477904

[26] Takehara K Sakamoto K Takahashi R Kawai M Kawano S Munakata S . Superior mesenteric artery syndrome improved by enteral nutritional therapy according to the controlling nutritional status score. Case Rep Gastroenterol. (2017) 11:729–35. doi: 10.1159/000484129, 29430225 PMC5803706

[27] Sun X Luo L Zhao X Ye P. Controlling nutritional status (CONUT) score as a predictor of all-cause mortality in elderly hypertensive patients: a prospective follow-up study. BMJ Open. (2017) 7:e015649. doi: 10.1136/bmjopen-2016-015649, 28928176 PMC5623525

[28] Miyata T Yamashita YI Higashi T Taki K Izumi D Kosumi K . The prognostic impact of controlling nutritional status (CONUT) in intrahepatic cholangiocarcinoma following curative hepatectomy: a retrospective single institution study. World J Surg. (2018) 42:1085–91. doi: 10.1007/s00268-017-4214-1, 28887635

[29] Shirakabe A Hata N Kobayashi N Okazaki H Matsushita M Shibata Y . The prognostic impact of malnutrition in patients with severely decompensated acute heart failure, as assessed using the prognostic nutritional index (PNI) and controlling nutritional status (CONUT) score. Heart Vessel. (2018) 33:134–44. doi: 10.1007/s00380-017-1034-z, 28803356

[30] Niwano M. The survival prognosis of elderly undernourished inpatients admitted to the internal medical department of an emergency hospital as assessed using the nutritional screening tool CONUT (for CONtrolling NUTritional status). Nippon Ronen Igakkai Zasshi. (2017) 54:356–63. doi: 10.3143/geriatrics.54.356, 28855460

[31] Mirili C Yılmaz A Demirkan S Bilici M Basol TS. Clinical significance of prognostic nutritional index (PNI) in malignant melanoma. Int J Clin Oncol. (2019) 24:1301–10. doi: 10.1007/s10147-019-01461-7, 31073814

[32] Onodera T Goseki N Kosaki G. Prognostic nutritional index in gastrointestinal surgery of malnourished cancer patients. Nihon Geka Gakkai Zasshi. (1984) 85:1001–5. 6438478

[33] Mureșan AV Hălmaciu I Arbănași EM Kaller R Arbănași EM Budișcă OA . Prognostic nutritional index, controlling nutritional status (CONUT) score, and inflammatory biomarkers as predictors of deep vein thrombosis, acute pulmonary embolism, and mortality in COVID-19 patients. Diagnostics. (2022) 12:12. doi: 10.3390/diagnostics12112757, 36428817 PMC9689150

[34] Hung KC Ko CC Wang LK Liu PH Chen IW Huang YT . Association of prognostic nutritional index with severity and mortality of hospitalized patients with COVID-19: a systematic review and meta-analysis. Diagnostics. (2022) 12:1515. doi: 10.3390/diagnostics12071515, 35885421 PMC9322949

[35] Zhang S Wang H Chen S Cai S Zhou S Wang C . Prognostic nutritional index and prognosis of patients with coronary artery disease: a systematic review and meta-analysis. Front Nutr. (2023) 10:1114053. doi: 10.3389/fnut.2023.1114053, 37006923 PMC10061069

[36] Kinney JM Elwyn DH. Protein metabolism and injury. Annu Rev Nutr. (1983) 3:433–66. doi: 10.1146/annurev.nu.03.070183.002245, 6357241

[37] Eckart A Struja T Kutz A Baumgartner A Baumgartner T Zurfluh S . Relationship of nutritional status, inflammation, and serum albumin levels during acute illness: a prospective study. Am J Med. (2020) 133:713–22.e7. doi: 10.1016/j.amjmed.2019.10.031, 31751531

