# Supplementary material for: Admission nutritional-immunological indices and sepsis risk in emergency trauma patients: a retrospective cohort study
Source: Front Nutr. 2026 Jun 10;13:1749592. doi: 10.3389/fnut.2026.1749592 (PMC13290438; doi:10.3389/fnut.2026.1749592)
Supplement: Supplementary file 1 [file Data_Sheet_1.PDF]

Table S1. Univariate regression analysis.

Variable	OR (95%CI)	*P* value
Age	1.03 (1.02~1.04)	< 0.001
Female	0.67 (0.47~0.96)	0.029
ISS	1.13 (1.12~1.15)	< 0.001
GCS	0.72 (0.68~0.76)	< 0.001
SOFA	1.67 (1.55~1.81)	< 0.001
WBC (×10^9/L)	1.1 (1.05~1.14)	< 0.001
Hb (g/L）	0.96 (0.95~0.97)	< 0.001
PLT(×10^9/L)	0.99 (0.98~0.99)	< 0.001
CR (μmol/L)	1.02 (1.01~1.03)	< 0.001
GLU (mmol/L)	1.07 (1.02~1.12)	0.003
PT (s)	1.16 (1.07~1.25)	< 0.001
D-Dimmer (μg/ml)	1.06 (1.04~1.08)	< 0.001
Diabetes n (%)	0.9 (0.53~1.52)	0.69
VENT (%)	54.65 (33.93~88.02)	< 0.001
EM operation (%)	4.74 (3.14~7.16)	< 0.001
LIVER (%)	0.99 (0.69~1.42)	0.962
DVT (%)	0.92 (0.66~1.27)	0.601
AKI (%)	0.86 (0.51~1.45)	0.57
AF (%)	0.82 (0.49~1.35)	0.434
Pneumonia1 (%)	0.91 (0.66~1.28)	0.599
RF (%)	1.03 (0.71~1.48)	0.893
CONUT	1.46 (1.38~1.55)	< 0.001
Mild risk	14.36 (3.43~60.06)	< 0.001
Moderate risk	100.58 (24.17~418.54)	< 0.001
Severe risk	130.27 (30.86~550.01)	< 0.001
PNI	0.85 (0.82~0.87)	< 0.001
PNI (≥ 39)	0.08 (0.05~0.11)	< 0.001

OR,odds ratio; CI, confidence interval; ISS,injury severity score; GCS, glasgow coma scale; SOFA, sequential organ failure assessment; Hb, hemoglobin; PLT, platelet; GLU, glucose; Cr, creatinine; PT, prothrombin time; EM operation, emergency operation; VENT, Mechanical ventilation; LIVER, chronic liver disease; DVT, deep venous thrombosis; AKI, acute kidney injury; HF, heart failure; RF, respiratory failure; CONUT, controlling nutritional status; PNI, prognostic nutritional index.

Table S2. The association between (CONUT score and PNI) and the incidence of sepsis, excluding patients with onset-to-admission time > 72 hours.

Variables	Unadjusted		Model 3	
	OR (95%CI)	*P*	OR (95%CI)	*P*
CONUT	1.44 (1.36~1.53)	< 0.001	1.17 (1.05~1.31)	0.005
PNI	0.85 (0.83~0.87)	< 0.001	0.89 (0.85~0.94)	< 0.001

Model 3: Adjusted for age, sex, ISS, SOFA, GCS, WBC, Hb, PLT, GLU, CR, PT, D-D, VENT, EM operation, AKI, HF, RF, DVT, Pneumonia, Liver, diabetes.

Table S3. Association of CONUT score and PNI with the incidence of sepsis after excluding the SOFA covariate.

Variables	Unadjusted		Model 3	
	OR (95%CI)	*P*	OR (95%CI)	*P*
CONUT	1.44 (1.36~1.53)	< 0.001	1.2 (1.05~1.37)	0.009
PNI	0.85 (0.83~0.87)	< 0.001	0.88 (0.83~0.94)	< 0.001

Model 3: Adjusted for age, sex, ISS, GCS, WBC, Hb, PLT, GLU, CR, PT, D-D, VENT, EM operation, AKI, HF, RF, DVT, Pneumonia, Liver, diabetes.

Table S4. Association of CONUT score and PNI with incident sepsis after excluding comorbidities and complications.

Variables	Unadjusted		Model 3	
	OR (95%CI)	*P*	OR (95%CI)	*P*
CONUT	1.46	< 0.001	1.19 (1.04~1.36)	0.010
PNI	0.85	< 0.001	0.88 (0.83~0.94)	< 0.001

Model 3: Adjusted for age, sex, ISS, GCS, WBC, Hb, PLT, GLU, CR, PT, D-D, VENT, EM operation.

Table S5. Three-level CONUT variable (normal + mild, moderate, severe) and the incidence of sepsis after category merging.

Variables	Unadjusted		Model 3	
	OR (95%CI)	*P*	OR (95%CI)	*P*
CONUT				
Q1+Q2	1(Ref)		1(Ref)	
Q3	10.6 (6.91~16.26)	< 0.001	4.9 (2.94~8.15)	<0.001
Q4	12.83 (7.95~20.7)	< 0.001	2.73 (1.42~5.27)	0.003
P for trend		< 0.001		<0.001

Model 3: Adjusted for age, sex, ISS, SOFA, GCS, WBC, Hb, PLT, GLU, CR, PT, D-D, VENT, EM operation, AKI, HF, RF, DVT, Pneumonia, Liver, diabetes.

Figure S1. Bootstrap internal validation (100 repetitions) of ROC curves for CONUT and PNI.

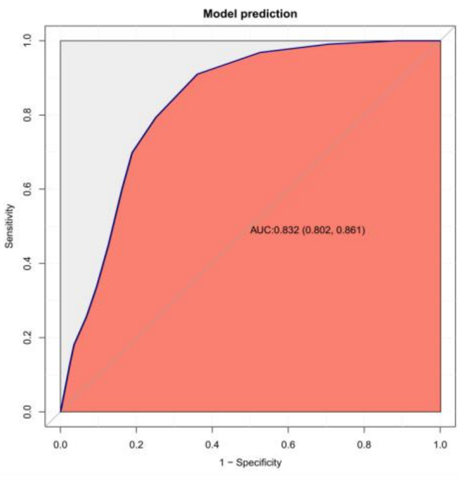

CONUT

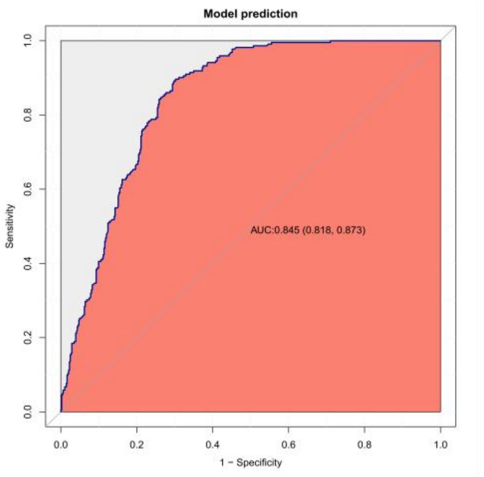

PNI
